# ECG-Facilitated Detection of Light Chain Cardiac Amyloidosis in Long-Standing MGUS

**DOI:** 10.1016/j.jaccas.2026.107622

**Published:** 2026-05-20

**Authors:** Jakub Piwowarski, Gina Barzen, Stephan Bohl, Katrin Hahn, Sebastian Spethmann

**Affiliations:** a Department of Cardiology, Angiology and Intensive Care Medicine, Deutsches Herzzentrum der Charité (DHZC), Campus Charité Mitte, Berlin, Germany; b Charité–Universitätsmedizin Berlin, Corporate Member of Freie Universität Berlin and Humboldt-Universität zu Berlin, Campus Charité Mitte, Berlin, Germany; c Amyloidosis Center Charité Berlin (ACCB), Amyloidosis Center of Charité, Campus Charité Mitte, Campus Benjamin Franklin, and Campus Virchow Klinikum, Berlin, Germany; d DZHK (German Centre for Cardiovascular Research), Partner Site Berlin, Berlin, Germany; e Department of Hematology, Oncology and Cancer Immunology, Charité–Universitätsmedizin Berlin, Corporate Member of Freie Universität Berlin and Humboldt-Universität zu Berlin, Campus Benjamin Franklin, Berlin, Germany; f Department of Neurology and Experimental Neurology, Charité–Universitätsmedizin Berlin, Corporate Member of Freie Universität Berlin and Humboldt-Universität zu Berlin, Campus Benjamin Franklin, Berlin, Germany; g Berlin Institute of Health at Charité–Universitätsmedizin Berlin, Berlin, Germany

**Keywords:** cardiac risk, electrocardiogram, risk factor, secondary prevention

## Abstract

A 76-year-old outpatient with light-chain monoclonal gammopathy of undetermined significance presented for evaluation of new-onset, nonspecific symptoms. Paraproteinemia remained stable for over 25 years without prior signs of organ manifestation. Physical examination was unremarkable. To complete routine diagnostics, a 12-lead electrocardiogram was performed, demonstrating features suggestive of amyloidosis, including low QRS voltages and an unexplained anterior pseudoinfarction pattern. It contributed to initiating further evaluation, leading to the diagnosis of light-chain cardiac amyloidosis.

**Take-Home Message:**

This case highlights the potential of electrocardiography as an easily accessible tool for increasing suspicion of cardiac complications in long-standing monoclonal gammopathy of undetermined significance, even in clinically inconclusive cases.

## Visual Summary

**Figure:**
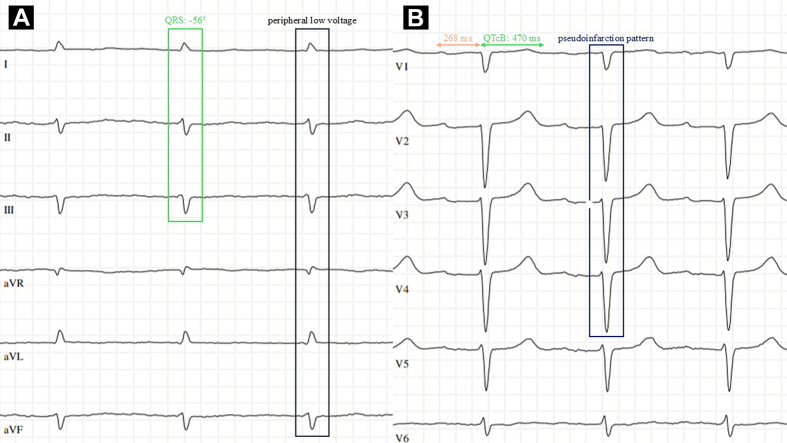

## Case Presentation

A 76-year-old man with long-standing monoclonal gammopathy of undetermined significance (MGUS), IgG lambda, presented to the outpatient medical clinic for evaluation of new-onset dizziness and mild exertional dyspnea. Paraproteinemia has been under regular hematologic-oncologic observation for over 25 years and showed no signs of organ involvement. Apart from recently diagnosed paroxysmal atrial fibrillation, the patient was free of cardiovascular disease. The physical examination was unremarkable, and anginal or syncopal symptoms were denied. To complete routine diagnostics, a 12-lead electrocardiogram (ECG) of the patient was performed.

**Visual Summary:**
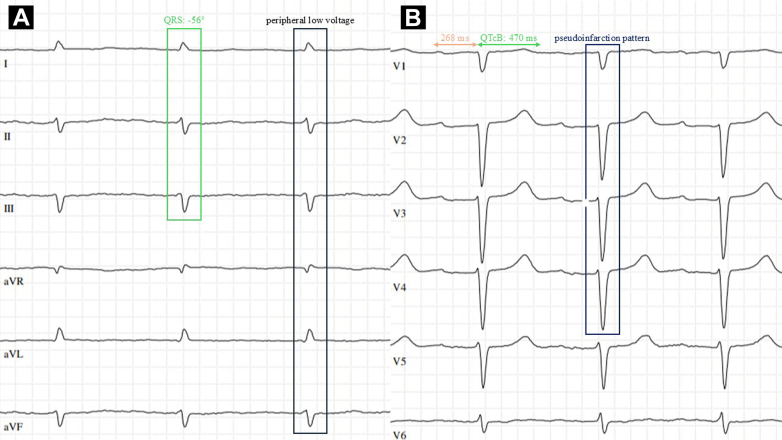
Typical Findings of Cardiac Amyloidosis on the Electrocardiogram Paper speed 50 mm/s, 1 mm = 0.1 mV. (A) Limb leads, (B) precordial leads. 1) Black box: absolute low voltage in peripheral leads. 2) Green box and arrow: left anterior fascicular block and QT interval prolongation, suggestive of the affection of the ventricular part of the conduction system. 3) Tuna arrow: first-degree atrioventricular block, suggestive of the affection of the atrioventricular part of the conduction system. 4) Blue box: pseudoinfarction pattern in anterior leads.

The ECG revealed nonspecific, diffuse conduction disturbances without correlating cardiovascular disease. Findings included first-degree atrioventricular block, left anterior fascicular block, and QT interval prolongation. Moreover, an unexplained anterior pseudoinfarction pattern was present despite no signs of coronary artery disease. Most notably, absolute low voltage in limb leads was observed. In this constellation, suspicion of cardiac amyloidosis (CA) was triggered, leading to the initiation of further diagnostic evaluation.

Because ECG findings in CA are frequently nonspecific and the differential diagnosis is broad, interpretation must be guided by the individualized clinical context. In this case, the absence of alternative etiologies and the patient's long-standing exposure to free light chains (Supplemental Table 1) made CA the most plausible explanation for the observed abnormalities. This was further supported by subsequent multimodality imaging, where no signs of coronary artery disease or competing infiltrative processes and cardiomyopathies were found.

Transthoracic echocardiography revealed a moderately thickened left ventricle (14 mm), biatrial enlargement, circular pericardial effusion, mildly impaired biventricular systolic function, grade II diastolic dysfunction, and an apical sparing pattern. Cardiovascular magnetic resonance further reinforced the suspicion, showing increased native T1 values and extracellular volume (39%), as well as diffuse subendocardial-to-midventricular late gadolinium enhancement without an ischemic pattern. Subsequently, technetium-99m-labelled 3,3-diphosphono-1,2-propanodicarboxylic acid scintigraphy, whole-body low-dose computed tomography, and rectal biopsy were scheduled, which revealed myocardial tracer uptake (Perugini grade III) but no osseous lesions and absent amyloid deposition in the obtained colon material. In turn, an endomyocardial biopsy was performed, revealing a diffuse amyloid deposition of the AL subtype. Consequently, the diagnosis of AL-CA was made, followed by the interdisciplinary disease management.

MGUS is a common chronic disorder that requires long-term follow-up^1^ and continuous vigilance for cardiac complications,^2^ even after decades of apparent stabilization. Therefore, widely available examinations are needed to support risk assessment in broader healthcare settings. This case highlights the potential role of ECG as one of such low-threshold tools to raise suspicion of new-onset AL-CA in long-established MGUS, even in outpatient care. Although evidence supporting routine ECG screening in MGUS is limited, this case suggests that ECG may serve as a useful adjunct to guideline-recommended multimodality imaging and biomarker monitoring. In particular, ECG evaluation could be particularly helpful when new symptoms arise, biomarker status deteriorates, or periodically in high-risk MGUS profiles. In turn, it could potentially contribute to detecting cases, which would otherwise be missed. However, these observations are hypothesis-generating and warrant further investigation. Finally, this case underscores the importance of adhering to guideline-directed diagnostic pathways to achieve the definitive detection of CA.^3^

### Patient Consent

The patient provided informed consent for the use of his data in scientific research and publications. This report adheres to the ethical standards outlined by the Committee on Publication Ethics.

## Funding Support and Author Disclosures

Dr Barzen received lecture fees, research funding, and travel reimbursement from Alnylam Pharmaceuticals, Intellia Therapeutics, and AstraZeneca. Dr Hahn received financial reimbursement for consulting, advisory board activities, speaker fees, and/or contributions to congresses and travel support to attend scientific meetings by Akcea Therapeutics Inc, Alnylam Pharmaceuticals Inc, Amicus, AstraZeneca, Bayer Vital GmbH, BridgeBio, GSK, Hormosan, Takeda Pharmaceutical Inc, Pfizer Pharmaceuticals Inc, Purpose Pharma, Swedish Orphan Biovitrum Inc, and ViiV Healthcare GmbH. Dr Hahn further received research funding from the Foundation Charité (BIH clinical fellow and digital accelerator), Alnylam Pharmaceuticals Inc, Amicus, AstraZeneca, Hormosan, and Pfizer Pharmaceuticals. None of these were related to this project. Dr Spethmann received consulting fees, advisory board fees, lecture fees, research funding, and travel grants for participation in scientific meetings from Pfizer Pharma GmbH, Apple Inc, Alnylam Netherlands BV, Bayer Vital GmbH, Boehringer Ingelheim Pharma GmbH, Edwards Lifesciences, Sanofi-Aventis Deutschland GmbH, Novartis Pharma GmbH, and AstraZeneca GmbH, which were not related to this project. All other authors have reported that they have no relationships relevant to the contents of this paper to disclose.
