# Supplementary material for: ECG-Facilitated Detection of Light Chain Cardiac Amyloidosis in Long-Standing MGUS
Source: JACC Case Rep. 2026 May 20;31(20):107622. doi: 10.1016/j.jaccas.2026.107622 (PMC13198135; doi:10.1016/j.jaccas.2026.107622)
Supplement: Supplemental Table 1 [file mmc1.docx]

Supplementary Table 1:
Levels of hematologic and cardiac biomarkers at the time of diagnosis

| *Hematologic Biomarkers: Serum* | | |
| --- | --- | --- |
| Biomarker | Concentration | Reference Range |
| Free lambda light chains | 111.05 mg/L | 5.70-26.30 mg/L |
| Free kappa light chains | 11.39 mg/L | 3.30-19.40 mg/L |
| Free kappa/free lambda ratio | 0.10 | 0.26-1.65 |
| Immunofixation electrophoresis | Precipitations against IgG and lambda antiserum | |
| *Hematologic Biomarkers: Urine* | | |
| Biomarker | Concentration | Reference Range |
| Free lambda light chains | 80.6 mg/L | < 5.0 mg/L |
| Free kappa light chains | 14.8 mg/L | < 8.0 mg/L |
| Free kappa/free lambda ratio | 0.18 | 0.80-4.50 |
| *Cardiac Biomarkers: Serum* | | |
| NT-proBNP | 3979 ng/L | < 592.0 ng/L |
| High-sensitivity troponin T | 23 ng/L | < 14.0 ng/L |

2012 Revised Mayo Clinic stage at diagnosis: II
